# Oral Colon-Targeted Lipid Nanoparticles Enhance Upadacitinib Delivery and Efficacy in a Murine Model of Ulcerative Colitis

**DOI:** 10.3390/ijms27093758

**Published:** 2026-04-23

**Authors:** Rabeya Jafrin Mow, Xiaodi Shi, Wen Lu, Siming Wang, Didier Merlin, Chunhua Yang

**Affiliations:** 1 Digestive Disease Research Group, Institute for Biomedical Sciences, Georgia State University, Atlanta, GA 30303, USA; rmow1@student.gsu.edu (R.J.M.); xshi10@student.gsu.edu (X.S.); dmerlin@gsu.edu (D.M.); 2 Department of Chemistry, College of Arts & Sciences, Georgia State University, Atlanta, GA 30303, USA; wlu@gsu.edu (W.L.); swang@gsu.edu (S.W.); 3 Atlanta Veterans Affairs Medical Center, Decatur, GA 30302, USA

**Keywords:** JAK inhibitor, bio-inspired lipid formulation, oral lipid nanoparticle, targeted drug delivery, inflammatory bowel disease (IBD)

## Abstract

Ulcerative colitis (UC) is a chronic inflammatory disorder of the colon characterized by dysregulated mucosal immunity and progressive epithelial injury. Upadacitinib (UPA), a selective Janus kinase 1 (JAK1) inhibitor, has demonstrated clinical efficacy in UC, but its therapeutic application is often constrained by adverse effects arising from systemic drug exposure. This underscores the need for advanced, site-specific delivery systems that enhance local efficacy while minimizing systemic toxicity. Here, we developed a colon-targeted natural lipid nanoparticle formulation of UPA (UPA-nLNP) to improve therapeutic performance and safety. UPA-nLNP was prepared by thin-film hydration using digalactosyldiacylglycerol (DGDG), monogalactosyldiacylglycerol (MGDG), and phosphatidic acid (PA), mimicking the lipid composition of ginger-derived exosomal particles, and was characterized for particle size, surface charge, and encapsulation efficiency. The formulation exhibited excellent mucus-penetrating capability and was evaluated in a dextran sulfate sodium (DSS)-induced acute colitis model in C57BL/6 mice following oral administration (5 mg/kg). Pharmacokinetic analysis demonstrated increased colonic accumulation with reduced systemic exposure compared to free UPA. Treatment with UPA-nLNP improved body weight recovery, reduced disease biomarkers, and suppressed key proinflammatory cytokines in the colon, with no evidence of systemic toxicity. This innovative strategy holds strong potential to enhance the clinical utility of JAK1 inhibitors by providing a safer and more effective therapeutic approach for ulcerative colitis.

## 1. Introduction

Inflammatory bowel disease (IBD) encompasses a group of chronic inflammatory conditions of the gastrointestinal (GI) tract, primarily including ulcerative colitis (UC) and Crohn’s disease (CD) [1,2,3]. UC is characterized by persistent mucosal inflammation that originates in the rectum and extends proximally in a continuous manner throughout the colon [4,5]. Although the underlying etiology of UC remains unclear, current evidence suggests it emerges from a multifaceted interplay of genetic susceptibility, environmental factors, microbial dysbiosis, and disruption of intestinal epithelial barrier integrity [6,7]. These alterations culminate in an abnormal immune response that perpetuates chronic inflammation. Clinically, UC manifests as episodes of rectal bleeding, diarrhea, abdominal pain, urgency, and tenesmus, with periods of remission in between. The global burden of UC continues to rise, contributing to the growing prevalence of IBD, which affects over 7 million individuals worldwide and significantly impairs quality of life while increasing healthcare costs [2,8].

The therapeutic landscape for UC has evolved considerably, aiming to induce and maintain remission, restore mucosal integrity, and improve quality of life. Mild-to-moderate cases are typically managed with aminosalicylates and corticosteroids, whereas immunomodulators and biologic agents are reserved for more severe cases [9,10]. Biologic therapies, including tumor necrosis factor-α (TNF-α) inhibitors (e.g., infliximab, adalimumab), anti-integrin agents (e.g., vedolizumab), and IL-12/23 inhibitors (e.g., ustekinumab), have significantly improved clinical outcomes. However, their systemic immunosuppressive action is associated with adverse effects such as heightened infection risk, malignancy, and thromboembolic events, in addition to requiring parenteral administration [11,12]. These limitations have prompted the exploration of alternative therapeutic strategies that combine efficacy with a more favorable safety profile and route of administration.

Advances in understanding the immunopathogenesis of UC have facilitated the development of targeted small molecules such as Janus kinase (JAK) inhibitors. These oral agents modulate intracellular signaling cascades downstream of multiple cytokine receptors, effectively dampening pro-inflammatory pathways [13,14]. Tofacitinib, a pan-JAK inhibitor, was the first approved oral JAK inhibitor for UC but is associated with safety concerns, including herpes zoster reactivation, dyslipidemia, venous thromboembolism, major adverse cardiovascular events (MACE) and increased mortality risk [15,16,17]. These risks have driven the development of second-generation JAK inhibitors with greater selectivity. Upadacitinib (UPA), a reversible and selective JAK1 inhibitor, was designed to improve efficacy while reducing off-target effects. Unlike many first-line agents with non-specific mechanisms, UPA selectively attenuates key inflammatory cytokines, including IFN-γ, IL-6, IL-7, and IL-23, and has demonstrated strong clinical activity by inducing rapid remission and promoting mucosal healing even in patients with moderate-to-severe UC refractory to conventional therapy [18,19,20,21]. Despite its efficacy, the clinical use of UPA remains constrained by systemic adverse events, including serious infections, hyperlipidemia, neutropenia, and thromboembolic complications—largely driven by systemic exposure associated with conventional oral dosing [22,23,24]. These risks underscore a critical need for targeted drug delivery strategies that enhance colonic targeting while minimizing systemic exposure.

Local drug delivery systems for UC have emerged as a promising solution to these challenges. Colon-targeted delivery can enable high local drug concentrations at sites of inflammation while limiting absorption in the upper GI tract, thereby reducing systemic toxicity [25,26,27]. This approach has been explored using various colon-targeted formulations, including pH-sensitive coatings and biodegradable polymers, as well as advanced nanoscale delivery systems such as polymeric nanoparticles, micelles, and lipid-based nanoparticles [27,28,29,30,31]. While most of these systems offer advantages such as controlled release and improved drug stability, they are often limited by poor mucus penetration, structural instability, or concerns regarding long-term biocompatibility [32,33]. In particular, certain synthetic nanoparticle systems have been associated with potential risks of accumulation, local toxicity, and unintended immune activation within the GI tract [34,35]. Lipid nanoparticles (LNPs), however, offer distinct advantages owing to their excellent biocompatibility, stability, and versatility. LNPs protect encapsulated drugs from gastrointestinal degradation, promote mucosal adhesion, and enable controlled release within inflamed colonic tissues [36]. Their small size, surface charge, and tunable lipid composition facilitate diffusion through the mucin network and modulate electrostatic interactions with mucins, thereby enhancing cellular uptake and site-specific drug delivery, particularly within the compromised mucosal barrier characteristic of UC [37,38]. Notably, biomimetic lipid nanoparticles inspired by plant-derived exosomal vesicles have gained traction due to their natural origin, inherent safety, and unique ability to target inflamed intestinal tissues [39,40,41]. These systems inherit favorable biocompatibility while leveraging structural features that enhance cellular uptake and site-specific delivery in colitis models. Encapsulating poorly water-soluble molecules like UPA, which are susceptible to degradation in harsh GI environments, within LNPs can improve solubility, protect against enzymatic and acidic breakdown, and enable localized drug delivery to inflamed mucosa while reducing systemic exposure.

Building on this rationale, we developed an innovative oral, colon-targeted lipid nanoparticle (nLNP) formulation inspired by the inherent colon-targeting properties of ginger-derived exosomal nanoparticles (GDNPs). The colon-localizing capacity of GDNPs arises from their intrinsic structural and compositional features. These exosomal nanoparticles are naturally enriched with digalactosyldiacylglycerol (DGDG), monogalactosyldiacylglycerol (MGDG), and phosphatidic acid (PA) [42]. The long hydrophobic fatty acid chains of these lipids form tightly packed, acid-resistant bilayers that protect the nanoparticle core from gastric degradation and enzymatic hydrolysis in the upper GI tract [36,42]. The negative charge of PA further stabilizes them in intestinal fluids, enabling their intact delivery to the colon. Importantly, this lipid composition enables transient interactions with colonic mucin glycoproteins, promoting retention of nanoparticles at inflamed sites [43]. Additionally, inflamed mucosa often contains positively charged proteins such as eosinophil cationic protein and transferrin, which can electrostatically interact with negatively charged GDNPs, promoting selective retention within inflamed tissue despite the overall negative charge of colonic mucus [29,36]. In IBD, disruption of epithelial tight junctions increases intestinal permeability, allowing GDNPs to penetrate beyond the mucus layer into the lamina propria, where immune cells actively internalize them [44]. GDNPs are also highly compatible with mammalian cell membranes and exhibit low immunogenicity [45]. Leveraging these attributes, which we replicated in our nLNP formulation by incorporating the same GDNP-mimicking lipid composition, our nLNP demonstrated selective accumulation in inflamed colonic tissue and preferential uptake by colonic epithelial cells and macrophages in a murine colitis model [46,47]. The inflammation-responsive behavior of this delivery platform positions it as an ideal candidate for oral, colon-targeted therapies.

In this study, we hypothesized that encapsulating UPA within our nLNP would enhance colonic drug delivery, thereby improving its anti-inflammatory efficacy while minimizing systemic toxicity. To test this hypothesis, we engineered and characterized the UPA-loaded nLNP and evaluated their performance in a DSS-induced acute colitis model using C57BL/6 mice, which recapitulates key pathological features of human UC. Biodistribution studies were conducted to assess colonic targeting and systemic exposure, followed by evaluation of therapeutic efficacy using disease activity indices and inflammatory markers. These findings provide a compelling rationale for the use of colon-targeted lipid nanoparticles to optimize the therapeutic potential of JAK inhibitors in the treatment of UC.

## 2. Results

### 2.1. Upadacitinib (UPA) Is Efficiently Encapsulated into nLNP

**Figure 1:**
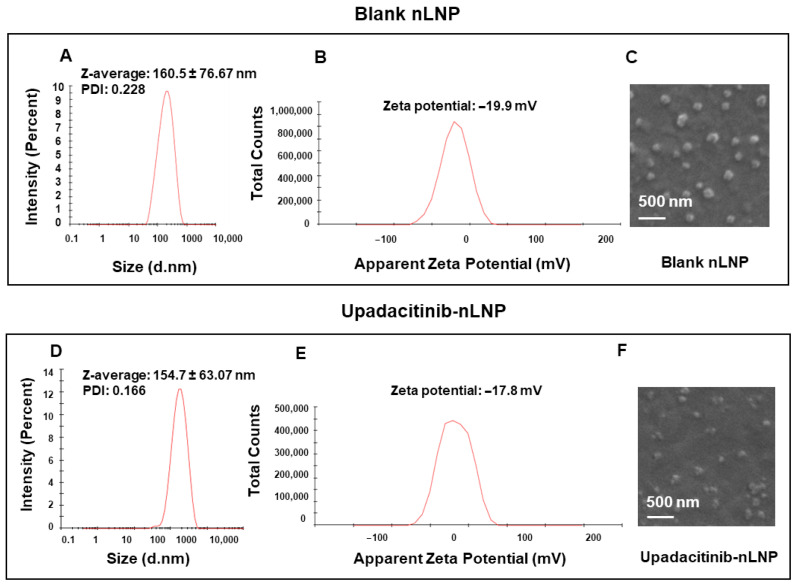
Characterization of blank nLNP and UPA-nLNP. (**A**) Dynamic light scattering (DLS) analysis showing the particle size distribution of blank nLNP. (**B**) Zeta potential measurement of blank nLNP. (**C**) Representative scanning electron microscopy (SEM) image illustrating the morphology of blank nLNP. (**D**) DLS analysis showing the particle size distribution of UPA-nLNP. (**E**) Zeta potential measurement of UPA-nLNP. (**F**) Representative SEM image showing the morphology of UPA-nLNP. All assays were performed in three independent batches, and data are presented as mean values.

Blank and UPA-loaded lipid nanoparticle were formulated using DGDG, MGDG, and PA in a 3:2:5 molar ratio to replicate the major lipid composition of GDNPs and thereby mimic their nanostructure and colon-targeting functionality [42,48]. Dynamic light scattering (DLS) revealed that the mean particle sizes of blank and drug-loaded nanoparticles were 160.5 ± 76.67 nm (Figure 1A) and 154.7 ± 63.07 nm (Figure 1D), respectively, indicating that drug encapsulation did not significantly alter particle size. The relatively large standard deviation for blank nLNP reflects some variability in particle size. The polydispersity index (PDI) values were 0.228 for nLNP and 0.166 for UPA-loaded lipid nanoparticle (UPA-nLNP), indicating a moderately uniform size distribution for nLNP and a narrow, highly uniform distribution for UPA-nLNP [49]. Zeta potential measurements showed negative surface charges of −19.9 mV (nLNP) (Figure 1B) and −17.8 mV (UPA-nLNP) (Figure 1E), consistent with the anionic nature of GDNPs and supporting colloidal stability by minimizing interparticle aggregation.

SEM confirmed a spherical morphology for both formulations (Figure 1C,F), further matching the structural characteristics of GDNPs. Collectively, the nanoscale size, negative surface charge, and spherical morphology validate that the nLNP closely resemble GDNPs in both composition and physicochemical properties, supporting their potential for efficient mucosal penetration and selective uptake in inflamed colonic tissues.

Furthermore, encapsulation efficiency study revealed a high encapsulation efficiency of 94.81% for upadacitinib in the nLNP formulation, underscoring the platform’s capacity for efficient drug loading. Collectively, these findings demonstrate that DGDG, MGDG, and PA can be reassembled into stable nanoparticles with favorable physicochemical properties for colon-targeted oral delivery of upadacitinib.

### 2.2. UPA-nLNP Maintains Stability Across Simulated Gastrointestinal Conditions

**Figure 2:**
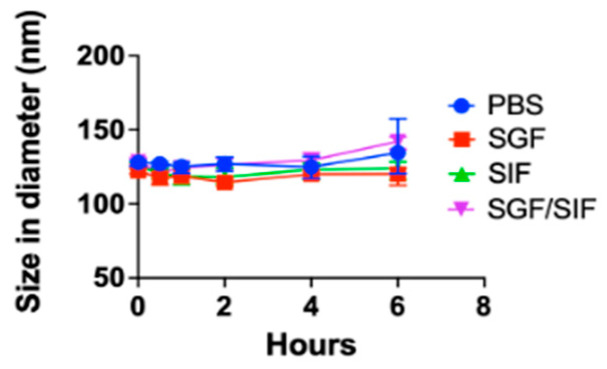
Stability study of UPA-nLNP in simulated gastrointestinal fluids. UPA-nLNP was incubated in PBS (pH 7.4), SGF (pH 3), SIF (pH 6.8), and a sequential SGF/SIF transition model at 37 °C. Particle size was measured over 6 h using DLS). Data are presented as mean (n = 3 independent batches).

The colloidal stability of UPA-nLNP was assessed in phosphate-buffered saline (PBS; pH 7.4, control), simulated gastric fluid (SGF; pH 3), simulated intestinal fluid (SIF; pH 6.8), and under a sequential simulated gastric-to-intestinal transition (SGF/SIF) model at 37 °C with agitation to simulate gastrointestinal transit. As shown in Figure 2, UPA-nLNP maintained stable particle size across all test conditions throughout the 6 h study. In PBS, SGF, SIF, and SGF/SIF, the nanoparticle size remained within the nanoscale range (~120–160 nm), with no signs of aggregation or colloidal instability.

These results confirm that UPA-nLNP retains its physicochemical integrity in both acidic and neutral environments, supporting its robustness for oral delivery and its potential for colon-targeted drug delivery.

### 2.3. UPA-nLNP Exhibits Enhanced Mucus Penetration

**Figure 3:**
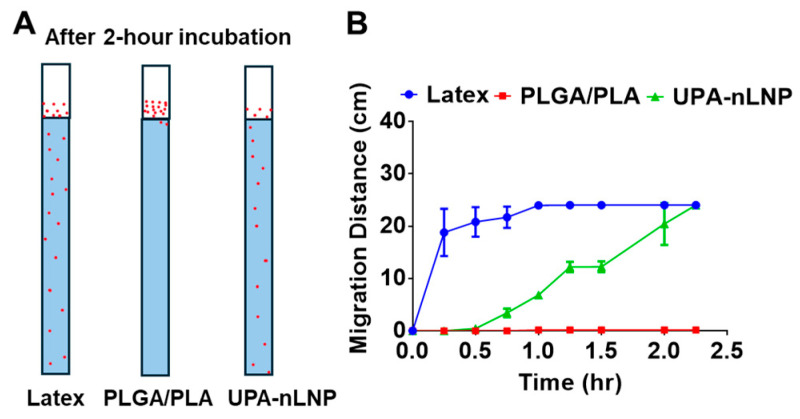
Mucus penetration behavior of nanoparticles in a 1% porcine mucin hydrogel. (**A**) Representative schematic illustrating the distribution of nanoparticles within the mucin column, including latex nanoparticles (positive control), PLGA/PLA nanoparticles (negative control), and UPA-nLNP. Red dots represent nanoparticles penetrating and distributing within the mucin matrix. (**B**) Quantitative analysis of nanoparticle migration distance over time (*n* = 3).

To evaluate the ability of UPA-nLNP to overcome biological barriers relevant to oral delivery, porcine mucin hydrogel, which closely mimics the physicochemical and rheological properties of intestinal mucus, was employed as a mucus-simulating system. As shown in Figure 3, latex nanoparticles (positive control) rapidly penetrated the mucin hydrogel, reaching the bottom of the column (~24 cm) within 1 h, whereas PLGA/PLA nanoparticles (negative control) exhibited negligible migration throughout the study period, indicating poor mucus permeability. In contrast, UPA-nLNP displayed a progressive, time-dependent increase in migration distance, ultimately reaching the bottom of the column at 2 h.

These findings demonstrate that UPA-nLNP possesses significantly enhanced mucus-penetrating capability compared to PLGA/PLA nanoparticles, approaching the behavior of the positive control. The mucus penetration behavior is likely attributable to the lipid-based composition and surface properties of UPA-nLNP, which facilitate diffusion through the mucin network. This supports the ability of UPA-nLNP to effectively traverse the intestinal mucus barrier, thereby enhancing localized drug delivery to inflamed colonic tissues.

### 2.4. UPA-nLNP Significantly Enhances Colonic Biodistribution of Upadacitinib While Minimizing Systemic Exposure

After confirming the stability and mucus-penetrating capability of UPA-nLNP, we next evaluated whether nLNP encapsulation enhances the colonic delivery of upadacitinib (UPA) using a pharmacokinetic (PK) study. An LC-MS/MS method with multiple reaction monitoring (MRM) was employed to quantify UPA concentrations in blood and colon tissues at multiple time points following oral administration of free UPA or UPA-nLNP (5 mg/kg) in C57BL/6 mice, using a sparse sampling design. Tissue exposure profiles were compared between the two treatment groups.

**Figure 4:**
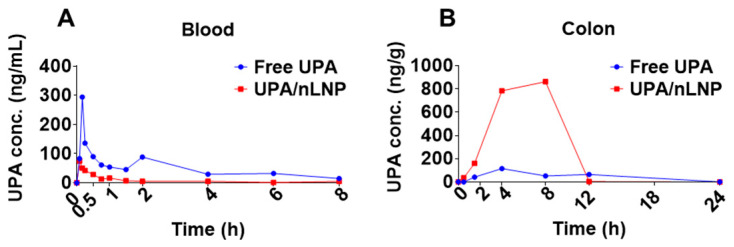
Pharmacokinetic profiles of Upadacitinib following oral administration of free UPA or UPA-nLNP. (**A**) Concentration–time profiles of UPA in blood. (**B**) Concentration–time profiles of UPA in colon tissue. Data are presented as mean (n = 3 per group; sparse sampling design).

As shown in Figure 4, the drug concentration–time profiles revealed distinct pharmacokinetic behaviors between the two formulations. In blood, free UPA exhibited rapid systemic absorption with a high peak concentration (Cmax: 300 ng/mL), whereas UPA-nLNP showed an approximately 4-fold lower Cmax (75 ng/mL) and an approximately 70% reduction in overall systemic exposure (AUC_0–8_ reduced from 485 ng·mL^−1^·h in the free UPA group to 146 ng·mL^−1^·h in the UPA-nLNP group), indicating effective limitation of systemic drug distribution (Figure 4A). The Tmax values were comparable between the two groups, suggesting similar initial absorption kinetics. In contrast, UPA-nLNP demonstrated markedly enhanced and sustained accumulation in colon tissues (Figure 4B). Specifically, UPA-nLNP achieved an approximately 7.5-fold higher Cmax (863 ng/g vs. 115 ng/g in free UPA) and an approximately 5-fold higher colonic exposure (AUC_0–24_ 6343 ng·g^−1^·h vs 1185 ng·g^−1^·h in free UPA), along with a delayed Tmax (8 h vs. 4 h), indicating prolonged retention and sustained drug release at the target site.

Collectively, these findings demonstrate that nLNP encapsulation significantly enhances the colonic drug exposure of UPA while minimizing systemic distribution. This favorable pharmacokinetic profile supports the ability of UPA-nLNP to achieve site-specific drug delivery, thereby potentially improving therapeutic efficacy while reducing the risk of systemic adverse effects.

### 2.5. Oral Colon-Targeted UPA-nLNP Therapy Accelerates Healing in Murine Colitis

**Figure 5:**
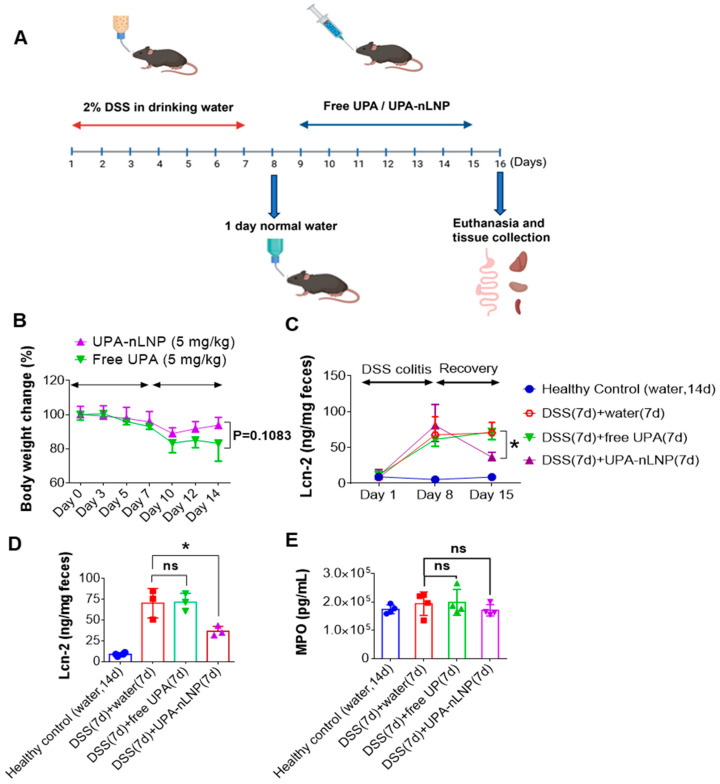
Oral administration of UPA-nLNP accelerates healing in murine colitis. (**A**) Experimental timeline of the DSS-induced colitis model and subsequent UPA-nLNP treatment. (**B**) Body weight changes during the study period. (**C**,**D**) Fecal lipocalin-2 (Lcn-2) levels at the indicated time points. (n = 3 per group; * *p* < 0.05; ns: not significant). (**E**) Colonic myeloperoxidase (MPO) activity. (n = 4 per group; ns: not significant).

We next investigated whether oral administration of UPA-loaded lipid nanoparticle (UPA-nLNP) could expedite the healing in a murine model of acute colitis. Female C57BL/6 mice were administered 2% DSS in drinking water for 7 days to induce acute colitis, followed by 1 day of regular water to reduce potential interference from residual DSS in the gastrointestinal tract (Figure 5A). Mice were then orally treated with UPA-nLNP or free UPA once daily for 7 days. Body weight was measured every two days. While the difference did not reach statistical significance (*p* = 0.1083), UPA-nLNP-treated mice demonstrated a consistent trend toward improved body weight recovery throughout the treatment period, suggesting a potential therapeutic benefit over free UPA in promoting clinical improvement during colitis recovery (Figure 5B). We also measured the fecal lipocalin-2 (Lcn-2) level, a sensitive and non-invasive biomarker of intestinal inflammation, to assess the anti-inflammatory effect of free UPA vs. UPA-nLNP [50,51]. It was significantly elevated in all DSS-treated groups after the 7-day induction phase. During the recovery period, Lcn-2 levels declined significantly in the UPA-nLNP-treated group by day 15, approaching those of healthy controls, whereas levels remained elevated in the free UPA group (Figure 5C,D). These data suggest that colon-targeted delivery of UPA via nLNP improves therapeutic efficacy and enhances recovery.

We further assessed colonic myeloperoxidase (MPO) activity as a measure of neutrophil infiltration and acute inflammation. Unexpectedly, MPO activity remained comparably high across all DSS-treated groups, including those treated with UPA-nLNP (Figure 5E). This lack of reduction in MPO, despite clear clinical improvement, may reflect the cell-specific targeting of nLNP, primarily toward colonic epithelial cells and macrophages—resulting in delayed neutrophil clearance from the tissue [46]. This is consistent with the notion that histological resolution often lags behind improvements in body weight and inflammatory biomarkers. Additionally, our cytokine analysis revealed a significant reduction in the pro-inflammatory cytokine IFN-γ only in the UPA-nLNP group (see Section 2.6), suggesting resolution at the molecular level even in the presence of residual cellular infiltrates. Together, these findings suggest that oral delivery of UPA-loaded nLNP markedly accelerates mucosal healing in a murine model of ulcerative colitis compared to free UPA.

### 2.6. Orally Administered UPA-nLNP Reduces Colonic Pro-Inflammatory Cytokine Expression

**Figure 6:**
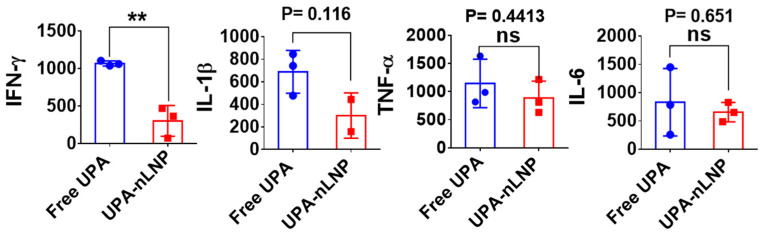
Colonic cytokine expression following oral administration of free UPA or UPA-nLNP. Mice with DSS-induced colitis received daily oral treatment with either free UPA (5 mg/kg) or UPA-nLNP (5 mg/kg). On day 16, colon tissues were harvested, homogenized, and analyzed for pro-inflammatory cytokines (IFN-γ, IL-1β, TNF-α, and IL-6) using Proteome Profiler Mouse Cytokine Array Kit. (n = 3 per group; ** *p* < 0.001; ns: not significant).

Cytokines play a pivotal role in orchestrating the onset, progression, and resolution of pathophysiological processes in ulcerative colitis (UC), with their local expression levels serving as critical biomarkers of disease activity. Among these, interferon-gamma (IFN-γ), interleukin-1 beta (IL-1β), interleukin-6 (IL-6), and tumor necrosis factor-alpha (TNF-α) are some key mediators of intestinal inflammation, contributing to epithelial damage, immune cell recruitment, and the amplification of downstream inflammatory cascades [52,53,54]. To assess the immunomodulatory impact of treatment, we quantified colonic expression of these cytokines following administration of either free UPA or UPA-nLNP. UPA is a selective JAK1 inhibitor that disrupts cytokine signaling pathways downstream of IFN-γ, IL-6, and IL-23 by inhibiting JAK1-dependent STAT phosphorylation [20,52]. This blockade attenuates the transcriptional activation of pro-inflammatory genes, particularly those regulated by the IFN-γ–JAK1–STAT1 axis, which is prominently implicated in UC pathogenesis [55]. As shown in Figure 6, oral administration of UPA-nLNP significantly reduced colonic IFN-γ expression relative to free UPA, indicating enhanced suppression of site-specific inflammation. IL-1β and TNF-α levels were also moderately decreased, though these reductions did not reach statistical significance, potentially due to inter-animal variability or the limited treatment duration.

Elevated IFN-γ level is known to trigger caspase-8–mediated Paneth cell apoptosis, impair crypt base columnar stem cell (CBC-ISC) survival, downregulate aquaporin-3 (AQP3), and upregulate claudin-2—cumulatively contributing to epithelial barrier disruption and impaired water absorption [19,56]. Its synergism with TNF-α further intensifies mucosal inflammation and epithelial damage [57,58]. Thus, the observed suppression of IFN-γ by UPA-nLNP likely contributes to enhanced mucosal protection and improved resolution of inflammation, even in the presence of residual TNF-α activity [20]. Collectively, these findings suggest that colon-targeted delivery of UPA via nLNP enables selective inhibition of the IFN-γ–JAK1–STAT1 axis, offering a promising strategy to modulate local immune responses while minimizing systemic exposure in the treatment of UC.

### 2.7. Oral Administration of UPA-nLNP Maintains Hematological and Biochemical Safety Profile

**Figure 7:**
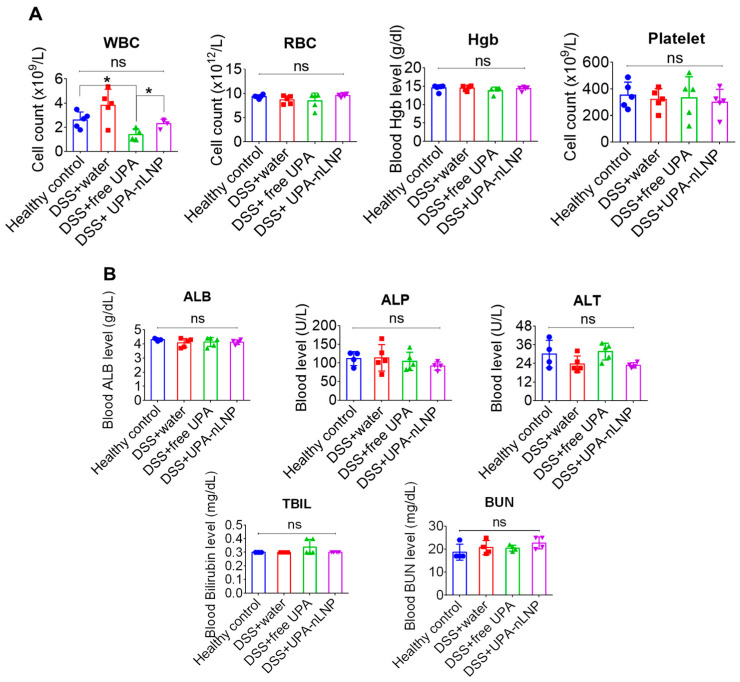
In vivo hematological and biochemical evaluation following oral administration of UPA-nLNP in DSS-induced colitis mice. (**A**) Blood samples were collected on day 16, and hematological parameters, including WBC (white blood cells), RBC (red blood cells), Hgb (hemoglobin), and platelet counts, were measured using an automatic hematology analyzer. (n = 5 per group) (**B**) Blood samples were analyzed for biochemical parameters, including ALB (albumin), ALP (alkaline phosphatase), ALT (alanine aminotransferase), TBIL (total bilirubin), and BUN (blood urea nitrogen) using a standard clinical chemistry analyzer. (n = 5 per group) (* *p* < 0.05; ns, not significant).

To evaluate the systemic safety of orally administered UPA-nLNP, key hematological and biochemical parameters were assessed using blood samples collected on day 16. As shown in Figure 7A, white blood cell (WBC) counts were significantly reduced in the DSS + free UPA group compared to healthy controls, suggesting potential immunosuppressive effects associated with systemic exposure to free UPA. In contrast, WBC levels in the DSS + UPA-nLNP group remained statistically comparable to healthy mice, indicating that colon-targeted delivery via nLNP preserves systemic immune cell populations. This preservation of peripheral WBC count is particularly favorable, as excessive leukopenia could impair host defense mechanisms and exacerbate vulnerability to infections, an important safety consideration in clinical translation.

Other hematological indices, including red blood cell (RBC) count, hemoglobin (Hgb) levels, and platelet count, showed no significant differences among the treatment groups, underscoring the overall hematologic safety of the nanoparticle formulation (Figure 7A).

Furthermore, analysis of liver and kidney function biomarkers, including albumin (ALB), alkaline phosphatase (ALP), alanine aminotransferase (ALT), total bilirubin (TBIL), and blood urea nitrogen (BUN), revealed no significant alterations across all groups (Figure 7B). All measured values remained within physiological ranges, supporting the absence of hepatotoxicity or nephrotoxicity following oral UPA-nLNP administration. Collectively, these results demonstrate that UPA-nLNP offers a safer alternative to free UPA by mitigating off-target hematological suppression while maintaining normal liver and kidney function. This reinforces the systemic safety of the UPA-nLNP platform in the DSS-induced colitis model.

## 3. Discussion

Lipid nanoparticles (LNPs) have emerged as a transformative platform for targeted drug delivery, particularly in inflammatory diseases where localized action is critical [36,59,60,61]. By encapsulating bioactive agents in nanoscale lipid vesicles, LNPs enable preferential accumulation in diseased tissues, enhancing therapeutic efficacy while minimizing systemic exposure and off-target effects. This is especially relevant for immunosuppressive drugs, which often suffer from dose-limiting toxicities when administered systemically. In the context of ulcerative colitis (UC), a chronic inflammatory condition of the colon, LNPs offer a promising strategy to deliver potent therapeutics directly to the inflamed mucosa.

The present study demonstrates that encapsulating the JAK1 inhibitor upadacitinib (UPA) within a colon-targeted natural lipid nanoparticle (nLNP) significantly enhances therapeutic efficacy while improving safety in a murine model of UC. The UPA-nLNP formulation exhibited favorable physicochemical properties, including nanoscale size, high encapsulation efficiency, and a slightly negative surface charge. These features are particularly advantageous for GI delivery, as they reduce nonspecific adhesion to mucus components while facilitating diffusion across biological barriers and retention within inflamed tissues. Consistent with these characteristics, UPA-nLNP treatment resulted in superior therapeutic outcomes compared to free UPA, as evidenced by improved body weight recovery and attenuation of inflammatory biomarkers. Notably, the marked reduction in colonic IFN-γ levels, alongside more modest changes in IL-1β and IL-6, suggests selective and efficient modulation of the IFN-γ–JAK1 signaling axis, which plays a central role in UC pathogenesis. Importantly, hematological and biochemical analyses confirmed the absence of systemic toxicity, underscoring the safety advantage conferred by localized drug delivery.

This enhanced therapeutic performance is closely linked to the ability of UPA-nLNP to efficiently traverse the intestinal mucus barrier, a critical determinant of successful oral drug delivery in the gastrointestinal tract. The dense and viscoelastic mucin network typically restricts nanoparticle diffusion and limits epithelial access. In this context, the optimized physicochemical profile of UPA-nLNP enables rapid diffusion through the mucus layer while minimizing adhesive interactions that would otherwise hinder mobility. This behavior facilitates closer interaction with the epithelial surface and promotes localized drug uptake within inflamed colonic tissue.

Consistent with this transport behavior, pharmacokinetic and biodistribution analyses revealed that UPA-nLNP significantly increased colonic drug exposure while limiting systemic distribution compared to free UPA. The elevated colonic area under the curve and delayed time to maximum concentration indicate sustained local drug release and prolonged residence within the target tissue, thereby maintaining therapeutic drug levels at the site of inflammation. In parallel, reduced plasma concentrations reflect restricted systemic absorption, which is critical for minimizing off-target effects. This coordinated balance between enhanced local retention and reduced systemic exposure represents a key advancement in improving the therapeutic index of JAK inhibitors, whose clinical utility is often constrained by systemic immunosuppression and associated adverse events.

These findings further emphasize the broader potential of colon-targeted delivery strategies within the JAK inhibitor therapeutic landscape for UC. Although UPA has demonstrated robust clinical efficacy, its systemic administration is associated with adverse outcomes such as infections, thromboembolic complications, and malignancy risks, largely driven by widespread immunosuppression. Targeted delivery to the inflamed colon enables localized modulation of immune signaling pathways while minimizing systemic exposure, thereby effectively decoupling therapeutic efficacy from toxicity. This paradigm is supported by previous studies on colon-targeted nanoparticle systems, including tofacitinib and RNA-based therapeutics, which have demonstrated enhanced therapeutic indices and improved safety profiles [62].

This study also builds upon growing evidence supporting the utility of nLNP as colon-targeting nanocarriers [42]. These natural lipid-based platforms have been used successfully in delivering diverse therapeutic agents. For instance, IL-22 mRNA-loaded nLNP promoted mucosal repair in DSS models, and M13-loaded nLNP reduced inflammation and prevented colitis-associated cancer via microbiome modulation [48,63]. Though the therapeutic cargos differ, these platforms share key advantages, including improved stability, preferential colon accumulation, and limited systemic dispersion. Our findings contribute to this body of work by demonstrating that small-molecule JAK inhibitors can similarly benefit from biomimetic lipid-based delivery, thereby broadening the applicability of this platform.

Mechanistically, our data highlight the central role of the IFN-γ–JAK1–STAT1 axis in UC and how efficiently UPA-nLNP inhibits this pathway. IFN-γ exacerbates colonic inflammation by driving Paneth and stem cell apoptosis, downregulating AQP3, and disrupting epithelial junctions [19,20]. By significantly lowering colonic IFN-γ levels, UPA-nLNP likely disrupts this cascade and supports mucosal healing. Although IL-6, IL-1β, and TNF-α levels were not significantly reduced, downward trends of IL-6 and IL-1β indicate partial attenuation of broader inflammatory responses, suggesting that combination therapeutic strategies may further enhance efficacy in chronic or more severe disease settings. These findings support colon-targeted oral delivery of UPA as a safer and more effective therapeutic strategy for UC, enabling localized drug activity while minimizing systemic exposure and associated long-term adverse effects. Furthermore, oral administration improves patient compliance and eliminates the need for injectable therapies, thereby enhancing clinical applicability. The absence of detectable systemic toxicity in this study further supports the feasibility of this platform for sustained therapeutic use.

While our findings provide compelling evidence that oral colon-targeted delivery of UPA via natural lipid nanoparticles improves therapeutic efficacy and safety in UC, certain limitations should be acknowledged. The study was conducted in an acute DSS model, which may not fully replicate chronic or relapsing UC. Future work should include chronic and spontaneous colitis models to validate efficacy and investigate whether UPA-nLNP can prevent long-term complications such as fibrosis. Further dose optimization and more comprehensive colonic pharmacokinetic profiling are also needed to refine therapeutic indices. Finally, exploring synergistic therapies, such as co-loading UPA-nLNP with agents targeting the TNF-α pathway, IL-22, or microbiome-modulating agents, could help determine whether synergistic suppression of inflammatory mediators and enhanced efficacy in complex inflammatory settings can be achieved.

From a translational perspective, the thin-film hydration method used here can be adapted to large-scale manufacturing via high-pressure homogenization or microfluidization. For long-term stability, strategies such as lyophilization with cryoprotectants, incorporation of stabilizing excipients, and lipid composition optimization will be explored to preserve physicochemical integrity and drug retention over extended periods.

## 4. Materials and Methods

### 4.1. Materials

#### Chemicals

Digalactosyldiacylglycerol (DGDG), monogalactosyldiacylglycerol (MGDG), and phosphatidic acid (PA) were purchased from Avanti Polar Lipids (Birmingham, AL, USA). Ethanol (200 proof) was obtained from Decon Labs (King of Prussia, PA, USA). Dichloromethane (DCM) was acquired from Sigma-Aldrich (St. Louis, MO, USA). Phosphate-buffered saline (PBS) was obtained from Corning (Manassas, VA, USA). Dextran sodium sulfate (DSS; MW 36,000–50,000 Da) was purchased from MP Biomedicals, LLC (Santa Ana, CA, USA). Carboxymethylcellulose sodium (CMC-Na) was obtained from Sigma-Aldrich (St. Louis, MO, USA). Upadacitinib (C_17_H_19_F_3_N_6_O; MW 380.4) was obtained from AA Blocks LLC (San Diego, CA, USA), and tofacitinib (C_16_H_20_N_6_O; MW 312.4) was purchased from Sigma-Aldrich (St. Louis, MO, USA). HPLC-grade methanol, dichloromethane (DCM), and acetonitrile (ACN) were purchased from Fisher Scientific (Palatine, IL, USA). Formic acid (98%, LC-MS grade) was obtained from ThermoFisher Scientific (Waltham, MA, USA). Simulated gastric fluid (SGF, pH 3.0) was prepared in-house and simulated intestinal fluid (SIF, pH 6.8) were purchased from Fisher Scientific (Palatine, IL, USA). Ultrapure deionized water was supplied by a Milli-Q water system (Millipore, Bedford, MA, USA). All other reagents and solvents used were of analytical or HPLC grade.

### 4.2. Methods

#### 4.2.1. Preparation of Upadacitinib-Loaded Lipid Nanoparticle

Upadacitinib-loaded lipid nanoparticle (UPA-nLNP) and empty nanoparticle (blank nLNP) were prepared using the thin-film hydration method [46]. Stock solutions were prepared separately as follows: DGDG, 5 mg in 2.5 mL 200-proof ethanol (2 mg/mL); MGDG, 5 mg in 2.5 mL 200-proof ethanol (2 mg/mL). Phosphatidic acid (PA) was used as a commercial 10 mg/mL solution. The three lipids were then mixed in a molar ratio of 3:2:5 (DGDG:MGDG:PA) in a pear-shaped flask. This lipid composition and ratio were selected to replicate the major lipid species naturally found in ginger-derived nanoparticles (GDNPs), enabling the resulting nLNP to retain their characteristic nanostructure and colon-targeting capability [42,48]. Upadacitinib was added to the lipid mixture, followed by additional ethanol to ensure complete dissolution. The lipid–drug solution was subjected to rotary evaporation (55–60 °C, 50 Torr) to remove organic solvents and form a uniform thin film on the inner surface of the flask. The dried film was hydrated with phosphate-buffered saline (PBS, pH 7.4), and the resulting suspension was sonicated in an ultrasonic bath at 55–60 °C with intermittent pipetting for approximately 5 min. This temperature range is below the reported degradation threshold for UPA, thereby ensuring chemical stability while enabling efficient lipid mixing [64]. The appearance of a homogenized, translucent suspension indicated successful formation of UPA-nLNP. Blank nLNP were prepared using the same procedure without the addition of UPA. All nanoparticle suspensions were stored at 4 °C until further use.

#### 4.2.2. Characterization of Upadacitinib-Loaded Lipid Nanoparticle

nLNP and UPA-nLNP were characterized for mean particle size, polydispersity index (PDI), and zeta potential in phosphate-buffered saline (PBS, pH 7.4) at room temperature using dynamic light scattering (DLS) on a Zetasizer (Malvern Panalytical, Westborough, MA, USA). The final lipid concentration used for DLS analysis was approximately 0.1 mg/mL.

Nanoparticle morphology was assessed by scanning electron microscopy (SEM). Briefly, nanoparticle suspensions were air-dried for 3 h at room temperature, mounted onto SEM stubs, and sputter-coated with a ~15 nm gold layer using a Denton Vacuum Desk II coater (Moorestown, NJ, USA). Imaging was conducted at an accelerating voltage of 15 kV using a Tescan VEGA 3 scanning electron microscope (Tescan Analytics, Fuveau, France) at the Imaging Core Facility of Georgia State University (Atlanta, GA, USA).

#### 4.2.3. Measurement of Encapsulation Efficiency

The encapsulation efficiency (EE%) of UPA in nLNP was determined using a centrifugal filtration method with Amicon^®^ Ultra-4 centrifugal filters (Ultracel^®^—100K, Millipore, USA). Briefly, UPA-loaded nLNP suspensions (sample A) were added to the upper chamber of the filters and centrifuged at 5000× *g* for 10 min at 4 °C. After centrifugation, the retained nLNP in the upper chamber containing the encapsulated drug were collected (sample B). To release the encapsulated drug, the retained nLNP was treated with methanol to dissolve the lipid matrix. The mixture was vortexed and appropriately diluted with HPLC solvent (50% Methanol) prior to analysis. To determine the total amount of drug loaded in the nanoparticles, an aliquot of the original UPA-nLNP formulation (sample A) was processed in parallel. It was similarly treated with methanol, vortexed, and diluted with HPLC solvent (50% Methanol) before HPLC analysis. All measurements were performed in triplicate. Encapsulation efficiency was calculated using the following equation [65]:

*EE (%) = {Encapsulated drug (sample B)/Total drug (sample A)} × 100%*

#### 4.2.4. Stability of UPA-nLNP in Simulated Gastrointestinal Fluids

The physicochemical stability of UPA-nLNP was assessed under simulated gastrointestinal conditions to mimic oral transit through the stomach and small intestine. Formulations were incubated at 37 °C with continuous agitation in the following media: phosphate-buffered saline (PBS; pH 7.4), simulated gastric fluid (SGF; pH 3), simulated intestinal fluid (SIF; pH 6.7–6.9), and a sequential SGF-to-SIF transition model. The SGF/SIF group involved initial incubation of UPA-nLNP in SGF for 1 h, followed by transfer to SIF, simulating physiological passage from the stomach to the small intestine. These pH conditions were selected to reflect the acidic gastric environment (pH 1.5–3.5), the near-neutral pH of the small intestine (~6–7), and the colonic pH (~7–7.4) typically encountered during murine oral delivery. At predefined time points (0, 0.5, 1, 2, 4, and 6 h), aliquots were withdrawn, and particle size was measured using dynamic light scattering (DLS) to evaluate colloidal stability. Time intervals were chosen to span the estimated gastric residence (0–2 h) and intestinal transit periods (2–6 h) in mice [66].

#### 4.2.5. Mucus Penetration Assay

The mucus-penetrating capacity of nanoparticles was evaluated using a mucin-based diffusion model adapted from previous reports [67]. Porcine gastric mucin was dissolved in phosphate-buffered saline (PBS) to prepare a 1% (*w*/*v*) mucin hydrogel, which was then transferred into vertically oriented 5 mL tubes to form a uniform mucus column. Nanoparticle suspensions (100 μL) of carboxylate-modified latex nanoparticles (CML latex; positive control; ~100 nm in diameter), PLGA/PLA nanoparticles (negative control; ~120 nm in diameter), and UPA-loaded natural lipid nanoparticle (UPA-nLNP; ~120 nm in diameter) were gently applied to the surface of the mucin hydrogel. The tubes were maintained in an upright position at room temperature throughout the experiment to ensure consistent diffusion conditions. At predefined time points (0, 0.25, 0.5, 0.75, 1, 1.25, 1.5, and 2 h), nanoparticle migration was quantified by measuring the penetration depth from the hydrogel surface.

#### 4.2.6. Quantification of Upadacitinib in Blood and Colonic Tissue via LC-MS/MS

To evaluate the pharmacokinetic profiles of free and nanoparticle-encapsulated Upadacitinib, UPA concentrations were quantified in blood and colon tissues at multiple time points following oral administration. Female C57BL/6 mice (~8 weeks old) were obtained from Jackson Laboratory (Bar Harbor, ME, USA) and maintained under specific pathogen-free conditions with a 12 h light/dark cycle. After a one-week acclimation period, mice were randomly assigned to two groups (n = 3 per group) and administered a single oral gavage of either free UPA (5 mg/kg, dissolved in 50 mM citrate buffer; pH 4.0) or UPA-nLNP (5 mg/kg, dispersed in PBS) in a total volume of 200 μL. Blood and colon samples were collected at predefined time points post-administration to construct concentration–time profiles using a sparse sampling design. At each time point, a separate cohort of mice was euthanized (terminal sampling), and blood and colon tissues were immediately harvested for analysis. Blood was collected via retro-orbital bleeding prior to euthanasia in accordance with Institutional Animal Care and Use Committee (IACUC)-approved protocols (#A23033, Georgia State University). Colons were excised, rinsed with PBS, and stored at −80 °C until analysis.

Sample preparation was performed using a protein precipitation method. Briefly, 300 μL of blood was mixed with 600 μL of ice-cold acetonitrile containing tofacitinib (125 μg/mL) as an internal standard, vortexed for 30 s, incubated on ice for 30 min, and centrifuged at 13,000 rpm for 10 min at 4 °C. The resulting supernatant (100 μL) was transferred to HPLC vials, and 5 μL was injected into the LC–MS/MS system. Colon tissues were homogenized in PBS (1:5, *w*/*v*) using a Tissue Miser homogenizer (Fisher Scientific, USA) and processed using the same extraction protocol. Samples exceeding the upper limit of quantification (ULOQ) were diluted with blank plasma and re-extracted to ensure quantification within the validated range [68].

Quantitative analysis was performed using a Waters Xevo TQ-S micro triple quadrupole mass spectrometer (Waters Corporation, Milford, MA, USA) coupled with a Waters Acquity UPLC H-Class Plus system (Waters Corporation, Milford, MA, USA). Chromatographic separation was achieved on an Agilent ZORBAX SB-C18 column (50 × 2.1 mm, 5 μm; Agilent Technologies, Santa Clara, CA, USA) using a gradient elution of 0.1% formic acid in water (mobile phase A) and acetonitrile (mobile phase B), increasing from 10% to 90% B over 3.5 min, followed by re-equilibration. The flow rate was maintained at 300 μL/min with an injection volume of 5 μL. Detection was performed using electrospray ionization (ESI) in positive mode with optimized multiple reaction monitoring (MRM) transitions of *m*/*z* 381.03 → 256.03 for UPA and *m*/*z* 313.03 → 148.95 for tofacitinib (internal standard). Instrument parameters included a capillary voltage of 3.0 kV, desolvation temperature of 350 °C, source temperature of 120 °C, and nitrogen as both nebulizing and drying gas. The method was validated over a linear range of 2.0–2080 ng/mL and used to quantify UPA concentrations in blood and colon samples for pharmacokinetic analysis.

#### 4.2.7. Evaluation of the Efficacy of Free UPA vs. UPA-nLNP

Female C57BL/6 mice (6–8 weeks old; Jackson Laboratory, Bar Harbor, ME, USA) were housed under specific pathogen-free (SPF) conditions with a 12 h light/dark cycle and acclimatized for one week prior to experimentation. All animals were wild-type, had no prior procedures, and were in good health to ensure experimental consistency. Mice were randomly assigned to four groups (n = 5 per group): (1) Healthy control, (2) DSS + water, (3) DSS + free UPA (5 mg/kg), and (4) DSS + UPA-nLNP (5 mg/kg). To minimize potential confounding variables, cage positions were rotated daily, and all treatments and sample collections were performed at the same time of day to reduce environmental and circadian variability. Experimental acute colitis was induced by administering 2% (*w*/*v*) dextran sulfate sodium (DSS; MP Biomedicals, Santa Ana, CA, USA) in drinking water for 7 consecutive days in Groups 2–4. This acute DSS-induced colitis model is widely used to mimic epithelial barrier disruption and mucosal inflammation observed in human ulcerative colitis, in which pathological changes impair barrier integrity, increase intestinal permeability, and allow luminal antigens to penetrate the mucosa, triggering robust immune responses [69]. Following DSS-induced colitis, mice were provided a one-day recovery period on normal water prior to treatment initiation. Daily treatments were administered via oral gavage for 7 days: Group 1 received water, Group 2 received water, Group 3 received free UPA, and Group 4 received UPA-nLNP at a dose of 5 mg/kg in a 200 μL volume, a dose selected based on prior preclinical pharmacokinetic and biodistribution studies in rodents to reflect an equivalent therapeutic systemic exposure used in murine UC models [70]. Body weight was monitored, and fecal pellets were collected every 2 days and stored at −80 °C for downstream analysis. Mice were also monitored daily for signs of distress, including significant weight loss (>20%), lethargy, and changes in stool consistency. Animals meeting humane endpoint criteria were promptly euthanized to minimize suffering.

Mice were euthanized on Day 16, 24 h after the final treatment. Colons were excised, and their lengths and weights were measured. Spleens were also weighed, distal colon tissues and other organs (spleen, liver, kidney, heart, brain, small intestine) were collected and preserved at −80 °C for further inflammatory biomarker analysis. All animal experiments were performed in compliance with institutional ethical guidelines and approved by the Institutional Animal Care and Use Committee (IACUC, protocol #A23033) of Georgia State University (Atlanta, GA, USA).

##### Quantification of Fecal Lipocalin-2 (Lcn-2)

As a reliable and non-invasive indicator of colonic inflammation, fecal lipocalin-2 (Lcn-2) was measured and used as a primary outcome marker in this study [71]. Approximately 100 mg of pre-weighed frozen fecal samples were resuspended in 1 mL of phosphate-buffered saline (PBS) containing 0.1% Tween-20 and vortexed for 20 min to obtain a homogeneous suspension. The samples were then centrifuged at maximum speed for 10 min at 4 °C, and the clear supernatants were collected. Fecal Lcn-2 levels were quantified using a DuoSet Mouse Lcn-2 ELISA kit (R&D Systems), following the manufacturer’s instructions [68].

##### Colonic Myeloperoxidase (MPO) Assay

Colon tissues were homogenized in pre-chilled phosphate buffer (pH 7.4). The homogenates were then centrifuged at 14,000 rpm for 15 min at 4 °C, and the resulting supernatants were collected for MPO quantification. MPO concentrations were measured using Mouse Myeloperoxidase DuoSet ELISA kit (R&D Systems), following the manufacturer’s instructions. Briefly, standards and samples were added to a 96-well microplate pre-coated with capture antibody and incubated according to the protocol. After washing to remove unbound material, a biotinylated detection antibody was added, followed by streptavidin-HRP and substrate solution. The reaction was stopped with sulfuric acid, and optical density was measured at 450 nm using a microplate reader. MPO levels were calculated based on the standard curve generated using recombinant MPO standards and normalized to tissue weight.

##### Analysis of Colonic Cytokine Expression

Colon tissues were homogenized in pre-chilled potassium phosphate buffer (pH 7.4). The homogenates were centrifuged at 14,000 rpm for 15 min at 4 °C, and the resulting supernatants were collected for cytokine quantification. Colonic cytokine levels were measured using the Proteome Profiler Mouse Cytokine Array Kit (Panel A, R&D Systems), a membrane-based antibody array that enables parallel detection of multiple mouse cytokines and chemokines, following the manufacturer’s instructions. Briefly, 1 mL of each standard or sample was mixed with 0.5 mL of array buffer and combined with the reconstituted Mouse Cytokine Array Panel A Detection Antibody Cocktail. The mixtures were gently mixed and incubated at room temperature for 1 h. Samples were then added to a 4-well multi-dish containing pre-treated membranes and incubated overnight at 4 °C. After washing to remove unbound materials, a biotinylated detection antibody was added, followed by incubation with streptavidin–HRP and chemiluminescent substrate. Then, 1 mL of the prepared Chemi Reagent Mix was evenly applied to each membrane and carefully covered with the top sheet of a plastic protector. Air bubbles were gently removed by tapping the plate on a flat surface until no visible bubbles remained, ensuring even distribution before measurement. The membranes were incubated for 1 min, and chemiluminescent signals were visualized directly on the membranes. Signal intensities were quantified using QuickSpots software (version 25, Ideal Eyes Systems).

#### 4.2.8. Hematological and Biochemical Analysis of Blood

Mice were anesthetized using 5% isoflurane, and whole blood was collected from the retro-orbital sinus, followed by euthanasia in accordance with the approved animal protocol (IACUC protocol #23033) at Georgia State University (Atlanta, GA, USA). For hematological analysis, blood samples were collected into EDTA-K3–coated tubes (1.3 mL, K3E; Sarstedt AG & Co., Nümbrecht, Germany), while Li-heparin–coated tubes (1.3 mL, LH; Sarstedt AG & Co., Nümbrecht, Germany) were used for serum biochemical testing. Complete blood counts were performed using a VetScan HM5 automatic hematology analyzer (Abaxis, Union City, CA, USA) with 50–100 μL of fresh whole blood per sample. The following parameters were assessed: white blood cells (WBC), red blood cells (RBC), hemoglobin (HGB), and platelets (PLT).

For blood chemistry analysis, 100 μL of fresh whole blood was analyzed using the VetScan VS2 analyzer (Abaxis, Union City, CA, USA) equipped with a comprehensive diagnostic rotor (Abaxis Europe GmbH, Griesheim, Germany). The following serum biomarkers were quantified: albumin (ALB), alkaline phosphatase (ALP), alanine aminotransferase (ALT), total bilirubin (TBIL), and blood urea nitrogen (BUN).

#### 4.2.9. Statistical Analysis and Data Visualization

All experimental data were recorded and processed using Microsoft Excel 365. Statistical analyses and graphical visualizations were performed using GraphPad Prism version 9.01 (GraphPad Software, San Diego, CA, USA). All values represent biological replicates and are presented as mean ± standard deviation (SD), along with 95% confidence intervals (CI) where applicable. Outliers were identified and excluded using the built-in outlier detection function in GraphPad Prism with an alpha value of 0.05; the exclusion criteria were not defined a priori. Only data points identified as statistical outliers were excluded; no animals were removed from the analysis. Statistical comparisons between two groups were conducted using unpaired two-tailed Student’s *t*-tests, assuming normal distribution. A *p*-value < 0.05 was considered statistically significant. The following notation was used to indicate significance: ns (not significant), * *p* < 0.05, ** *p* < 0.01, *** *p* < 0.001.

## 5. Conclusions

This study demonstrates that oral colon-targeted delivery of Upadacitinib via natural lipid nanoparticle (UPA-nLNP) offers a promising strategy to enhance therapeutic efficacy while minimizing systemic toxicity in ulcerative colitis. The UPA-nLNP formulation effectively localized drug action to the inflamed colon, leading to significant suppression of IFN-γ and improved clinical outcomes in a murine colitis model. Importantly, hematological and biochemical analyses confirmed a favorable safety profile, supporting the translational potential of this platform. Collectively, these findings highlight the therapeutic potential of site-specific JAK1 inhibition and position lipid nanoparticle-based oral delivery as a compelling strategy to enhance the therapeutic index of small-molecule therapies for inflammatory bowel disease.

## Data Availability

The data underlying this article will be shared at reasonable request to the corresponding author.
